# Parent–Child Relationships and Locus of Control in Two-Child Families

**DOI:** 10.11621/pir.2026.0104

**Published:** 2026-03-01

**Authors:** Irina E. Rzhanova, Olga S. Alekseeva

**Affiliations:** a Federal Scientific Center for Psychological and Interdisciplinary Research, Moscow, Russia

**Keywords:** locus of control, parent-child relationships, siblings, family environment, Actor–Partner Interdependence Model (APIM)

## Abstract

**Background:**

In a rapidly changing society, locus of control becomes an important psychological indicator reflecting an individual’s readiness to actively manage their own life. Research shows that an internal locus of control is associated with higher levels of psychological well-being, academic achievement, and social responsibility. The relevance of studying the factors that influence the development of locus of control in childhood and adolescence is determined by its high plasticity during this period and the possibility of targeted influence through the family environment.

**Objective:**

The present study focuses on the relationship between parental and child locus of control in the context of parent–child relationships in intact two-child families. Particular attention is given to the analysis of both direct and indirect influences (through parental attitudes) of parental locus of control on children’s locus of control.

**Design:**

The sample consisted of 278 families (N = 1,112 individuals). The Questionnaire of Locus Control and the “Parent-Child Interaction” questionnaire were used for assessment. Data analysis was conducted using the Actor–Partner Interdependence Model (APIM).

**Results:**

Parents with a more internal locus of control demonstrated more positive attitudes toward their children, which in turn was positively associated with the development of an internal locus of control in siblings. Maternal locus of control was found to play a particularly important role, exerting both direct and indirect influences on children’s locus of control. Within the parental dyad, maternal influence was expressed not only as actor effects but also as partner effects, through its impact on paternal attitudes toward the children. It was also shown that children’s subjective perception of a parental positive attitude is a particularly significant mediator between parental and child locus of control.

**Conclusion:**

The obtained data emphasize the significance of the family environment, specifically positive parental attitude, in the formation of locus of control in children.

## Introduction

The concept of locus of control was introduced in 1966 and is a central construct of J. B. Rotter’s social learning theory (Nowicki et al., 2021; Rotter, 1966). In general terms, locus of control can be defined as a personality trait that reflects the tendency to attribute one’s successes and failures either to internal or to external factors. Locus of control is a bipolar characteristic. On one pole, the pole of internality, are individuals convinced that what happens to them depends on their personal qualities and that they are able to control their own fate. On the opposite pole, the pole of externality, are individuals who attribute everything that happens to them (successes and failures) to external factors such as fate, chance, or social environment. It is assumed that every person occupies a certain position on the continuum stretching from an external to an internal locus of control. The stability of locus of control as a psychological trait increases with age: longitudinal studies show high variability of this characteristic in childhood and low variability in adulthood (Nowicki et al., 2018).

Locus of control is associated with a wide range of psychological and behavioral characteristics. An internal locus of control is linked to higher levels of tolerance and responsibility (Ahlin & Antunes, 2015), greater self-control, stronger ability to cope with stressful situations, and better adaptation to new conditions (Zulkarnain et al., 2019). It has been shown that adolescents with an internal locus of control are less likely to be involved in violent activities and are more protected from various forms of destructive behavior (Ahlin & Antunes, 2015). Internal locus of control is positively associated with academic achievement (Au, 2015; Strayhorn, 2010) and with higher levels of various cognitive indicators (Anderson et al., 2018), and it is also a significant predictor of both mental and physical health (Botha & Dahmann, 2023; Kesavayuth et al., 2022). In contrast, an external locus of control is positively associated with a greater tendency toward aggressive behavior (Ahlin & Antunes, 2015; Wallace et al., 2012), lower self-esteem, and poorer ability to cope with stressful situations (Asberg & Renk, 2014). Individuals with an external locus of control are also more likely to experience anxiety and are more susceptible to depression (Costantini et al., 2021; Yu & Fan, 2016).

### Parent–Child Relationships and Locus of Control

Studies on the relationship between parental locus of control and children’s locus of control show mixed results. Some studies did not find significant within-pair correlations for this parameter (Becker et al., 2010), while others did report such associations (Chandler et al., 1980). A large longitudinal study involving 3,500 families in England found modest yet significant associations between parental and child locus of control, measured when the children reached the ages of eight and sixteen (Nowicki et al., 2018).

The influence of parental locus of control on parenting style has often been a subject of research interest (Jain & Lokesh, 2023; Yaffe, 2024). It is assumed that parents’ beliefs about their ability to significantly influence life events play an important role in the parenting strategies they use. Studies have shown that an external parental locus of control is associated with a tendency to use violence, particularly physical punishment, toward children (Bugental et al., 1989). A later study on the impact of parenting style on preschoolers’ behavioral problems, such as aggression and bullying, found that parents with an external locus of control were less consistent with their children and used punishment more frequently (Kokkinos & Panayiotou, 2007). In contrast, mothers with an internal locus of control, when solving tasks with their child, more often used supportive strategies: they smiled, expressed approval, and showed warmth regardless of the child’s success (Carton et al., 1996).

Of particular interest are the data on the relationship between parental (especially maternal) locus of control and children’s academic achievement, as well as their behavior at school. Maternal externality is a significant predictor of difficulties in acquiring mathematical knowledge by schoolchildren (Golding et al., 2019). Earlier, the same group of researchers found an effect of maternal locus of control on children’s intelligence scores, assessed at the ages of four and eight using the WISC (Golding et al., 2017). Parental locus of control, measured even before the birth of the child, predicted difficulties in school behavior: a prenatal external locus of control in parents was associated with aggression and with systematic failure to follow instructions when learning new material (Nowicki et al., 2018).

It is well known that the quality of family relationships is associated with many personality traits (Chursina, 2025; Huver et al., 2010; Park & Kim, 2024; Quang Dao & Le, 2025). Numerous studies have shown the significant role of the family environment and parental attitudes in the formation of a child’s locus of control. Parental warmth, acceptance, and involvement in children’s activities are associated with the development of an internal locus of control (Ahlin, 2014; Ahlin & Lobo Antunes, 2015). On the other hand, parental overcontrol and low levels of acceptance are associated with an external locus of control (Spokas & Heimberg, 2009). Overall, a stressful family environment is a substantial predictor of an external locus of control in children. For example, parental alcoholism and mental disorders (Yates et al., 1994), as well as physical punishment and child abuse (Roazzi et al., 2016), are linked to child externality.

Thus, a brief review of the literature shows that locus of control is closely interrelated to the characteristics of the family environment, especially to parameters of parent–child relationships. However, there is a lack of studies that investigate the dyadic influence of parental traits on children’s personality. The aim of the present study was to analyze the influence of parental locus of control on children’s locus of control, both directly and indirectly — mediated through its effect on parental attitudes toward children. A distinctive feature of this work is the application of the Actor–Partner Interdependence Model (APIM) for data analysis, which was used to assess the interrelationship between participants of the parental dyad on the given indicators. Actor–partner interdependence models were constructed for parental and child locus of control, with various parameters of parental attitudes toward children included as mediating variables.

## Methods

### Participants

The study involved 278 intact families with two children, with a total of 1,112 participants. The mean age of fathers was 45 years (SD = 5.28), mothers — 43 years (SD = 4.36), older children — 18 years (SD = 2.31), and younger children — 15 years (SD = 2.41). At the time of the study, all families were living in Moscow and the Moscow region.

### Procedure

To assess the locus of control of parents and children, the Questionnaire of Subjective Control consisting of 44 items was used (Bazhin et al., 1993).

To evaluate parental attitudes, the “Parent–Child Interaction” questionnaire was applied (Markovskaia, 2006). The questionnaire has two mirror forms, allowing for the collection of both parental and child assessments of the family relationships. A previous factor analysis of this questionnaire, conducted on a sufficiently large sample, showed discrepancies between the factor structures obtained in the parent group and in the child group. Statistical analysis of the parent version revealed five main factors: Parental Positive Attitude, Parental Control, Parental Gentleness, Parental Consistency, and Parent-Child Trust. Statistical analysis of the child version revealed three main factors: Parental Positive Attitude, Parental Control, and Democratic Attitude. All identified factors met validity and reliability criteria (Alekseeva & Kozlova, 2010). In this article, parent-child relationships were assessed using scores based on the factors described above.

### Data Processing Methods

The results were processed using the SPSS Statistics software package, version 18.0. Correlation analysis and paired-sample t-tests were applied. All models were estimated in EQS 6.3 using the ML method.

#### Actor–Partner Interdependence Model

In the present study, statistical modeling based on the Actor–Partner Interdependence Model (APIM) was applied to assess the relationships between members of the family dyad on the selected variables (Cook & Kenny, 2005; Egorova et al., 2022; Rzhanova, 2024). This type of statistical modeling is capable of revealing how a specific psychological characteristic of one person in a dyad influences certain parameters — both their own (actor effects) and their partner’s (partner effects). Moreover, APIM allows not only the identification of significant associations between variables, but also the examination of the direction of influence from one variable to another, suggesting causal links (Cook & Kenny, 2005; Kim & Kim, 2024). An additional advantage of this method is the ability to include mediating variables in the analysis.

## Results

### Differences and Similarities in Locus of Control within Family Dyads

**Figure 1.**
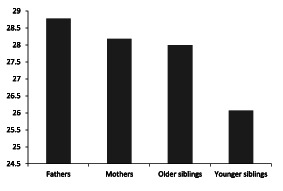
Mean locus of control scores among family members

Differences in locus of control scores within family dyads were assessed using Student’s t-test. *Figure 1* shows the mean locus of control scores for each family member. Significant differences on this parameter were found for the pairs father–younger sibling (*t* = 5.05, *p* < .01), mother–younger sibling (*t =* 3.64, *p <* .01), and older sibling–younger sibling (*t =* 3.59, *p <* .01). Overall, younger children demonstrated a lower level of locus of control compared to other family members.

### Correlation Between Parental Attitudes Toward Children and Locus of Control

**Figure 2.**
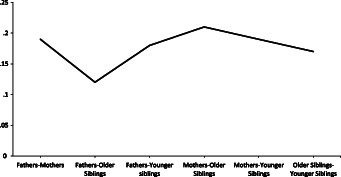
Within-pair similarity in locus of control

To analyze the relationships between parental attitudes and locus of control in different family members, we calculated correlations using Spearman’s nonparametric coefficient. In particular, within-pair correlations for locus of control in various family dyads were examined (see *Figure 2*). The correlation coefficients were positive and relatively low, reaching statistical significance in all dyads except for father–older child pairs.

In the course of further statistical data processing, correlations were found between locus of control and various parameters of parental attitudes. The strongest correlations with locus of control, both for parents and children, were demonstrated by the parameter Positive attitude toward the child. This association was observed in both the parental and child versions of the questionnaire. The parameter Positive attitude toward the child was obtained through factor analysis of the “Parent–Child Interaction” questionnaire on a fairly large sample of parents and children (Alekseeva & Kozlova, 2010). This parameter has the highest factor loading in both respondent groups and is generally consistent in psychological meaning across the parent and child versions. It reflects the level of parental acceptance and closeness with the child, overall satisfaction with parent–child relationships, as well as parental authority. *Table 1* presents the correlation coefficients between locus of control and Positive attitude toward the child, as assessed by different family members.

**Table 1:** Association of Locus of Control with the Parameter “Positive Attitude Toward the Child”

**Parental attitude parameters**	**Father LOC**	**Mother LOC**	**Older sibling LOC**	**Younger sibling LOC**
Father’s positive attitude toward older sibling — Parent rating	.21**	.21**	.11	.02
Father’s positive attitude toward younger sibling — Parent rating	.22**	.09	.03	.10
Mother’s positive attitude toward older sibling — Parent rating	.14*	.38**	.07	.12
Mother’s positive attitude toward younger sibling — Parent rating	.13*	.23**	.01	.16*
Father’s positive attitude toward older sibling — Child rating	.13*	.16*	.23**	.08
Father’s positive attitude toward younger sibling — Child rating	.25**	.14*	.10	.21**
Mother’s positive attitude toward older sibling — Child rating	.08	.24**	.18*	.03
Mother’s positive attitude toward younger sibling — Child rating	.10	.14*	.04	.28**

Note. * p < .05; ** p < .01.

The results indicate that the nature of the associations between locus of control and parental attitudes strongly depends on which family member provides the assessment. Overall, parental locus of control is positively related to both parental and child ratings of positive parental attitudes toward the child. Moreover, maternal locus of control shows more associations compared to paternal locus of control. The higher the level of locus of control in parents, the better their relationships with children are.

The locus of control of both siblings is positively associated with their own ratings of parent–child relationships rather than with parental ratings. Siblings with an internal locus of control tend to evaluate their parents’ attitude toward them as more positive.

### Analysis of the Relationships Between Locus of Control and Parental Attitudes Using Actor–Partner Modeling

**Figure 3.**
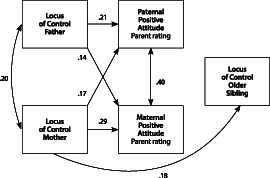
Actor–partner model: dependent variable — locus of control of the older sibling; mediating variable — parental rating of Positive attitude toward the child (χ^2^(2) = .722; CFI = 1.000; RMSEA = .000).

**Figure 4.**
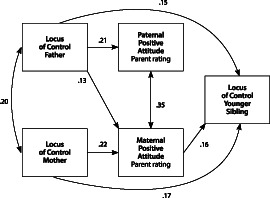
Actor–partner model: dependent variable — locus of control of the younger sibling; mediating variable — parental rating of Positive attitude toward the child (χ^2^(3) = 3.704; *CFI*= .992; *RMSEA* = .028).

Based on the results of the correlation analysis, strong relationships were found between parental and child locus of control and the “Positive Parental Attitude Toward the Child” parameter. This led us to hypothesize that this parameter may serve as a mediator through which parental locus of control influences the locus of control of siblings. To test this assumption, APIMs were constructed for both older and younger siblings, in which the parental rating of Positive attitude toward the child served as the mediator between parental and child locus of control (see *Figures 3 and 4*). Both models showed a good fit to the data. The corresponding figures are presented below. All links shown in the figures here and below are statistically significant.

The mother’s locus of control has a significant influence on her attitude toward the older child, and a similar, though less pronounced, association is observed for the father’s locus of control. Since a positive association was found between both parents’ locus of control and their own positive attitude toward the older sibling, we can conclude that significant actor effects of parental locus of control on this parameter of parent–child relationships were obtained. In addition to these actor effects, significant partner effects were also identified: the locus of control of both parents was positively related to the partner’s positive attitude toward the child. Thus, a dyad-oriented pattern of mutual influences of parental locus of control on positive attitudes toward the older sibling was revealed. However, the hypothesis of a mediating role of parental positive attitudes was not confirmed: both maternal and paternal locus of control were directly associated with the same trait in the older sibling.

The actor–partner model examining variables influencing the locus of control of the younger sibling differs from the model constructed for the older sibling (see *Figure 4*).

Significant positive actor effects of parental locus of control on positive attitudes toward the younger child were obtained. However, a significant partner effect was found only for paternal locus of control: it was associated with the mother’s positive attitude toward the younger sibling. Maternal locus of control did not show a partner effect on the father’s attitude. Nevertheless, maternal locus of control had a significant influence on the locus of control of the younger child: this association was both direct and indirect, through the mediating variable of maternal positive attitude toward the child. Paternal locus of control also exerted an indirect influence on the locus of control of the younger sibling, expressed as a partner effect, through its impact on maternal attitude.

**Figure 5.**
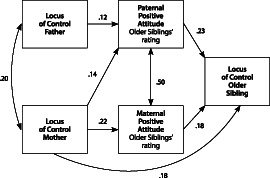
Actor–partner model: dependent variable — locus of control of the older sibling; mediating variable — child rating of positive parental attitude (χ^2^(2) = 2.470; *CFI* = .997; *RMSEA* = .025).

**Figure 6.**
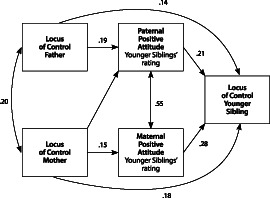
Actor–partner model: dependent variable — locus of control of the younger sibling; mediating variable — child rating of positive parental attitude (χ^2^(3) = 1.467; *CFI* = 1.000; *RMSEA* = .000).

Further APIMs were constructed in which sibling ratings of parental attitudes served as the mediators between parental and child locus of control (see *Figures 5 and 6*). Both models showed a good fit to the data.

The locus of control of both parents showed actor effects on how the older sibling evaluated parental attitudes. An internal locus of control in parents is associated with a higher assessment of the parental attitude. The influence of maternal locus of control on the locus of control of the older child was stronger than that of the father, as it was realized indirectly — through both actor and partner effects on parental positive attitude toward the child — as well as directly, through the association between maternal parameters and those of the older child (see *Figure 5*).

The APIM for the younger sibling’s ratings is generally similar to the corresponding model for the older sibling. Maternal locus of control exerts both direct and indirect influences on the locus of control of the younger child, with these effects being realized as both actor and partner effects (see *Figure 6*).

## Discussion

The study examined parental attitudes toward children and locus of control across two generations within the same family. The results demonstrate significant differences in locus of control identified across various family dyads. Younger children demonstrated lower levels of locus of control than other family members. This can be explained by age-related influences: the younger siblings who participated in the study were adolescents. It has been established that during childhood and adolescence, locus of control is less stable and more variable compared to older age groups (Nowicki et al., 2018). Within-pair similarities in locus of control across different family dyads were low but statistically significant, with the exception of father–older sibling pairs, where the correlations did not reach the level of statistical significance.

Parental locus of control is positively associated with ratings of positive attitudes toward children. Furthermore, the maternal indicator demonstrates more associations compared to the paternal one. Parents with a more internal locus of control tend to have better relationships with their children, both according to their own assessments and those of their children. Numerous previous studies have revealed a similar pattern (Bugental et al., 1989; Carton & Nowicki, 1996; Kokkinos & Panayiotou, 2007; Nowicki et al., 2018). This association may be because parents with an internal locus of control believe that their behavior and attitudes play a decisive role in the child’s psychological development and that they can actively shape family relationships. As a result, they strive to show greater warmth and acceptance and to monitor and reduce inconsistent or negative reactions toward their children.

The locus of control of siblings is positively correlated with their perceived positive parental attitudes. Children with a more internal locus of control tend to evaluate parental attitudes toward them as more positive compared to children with a more external locus of control. The influence of parental acceptance and warmth on child internality has been demonstrated previously (Ahlin, 2014; Ahlin & Lobo Antunes, 2015). However, a distinctive feature of the present study is that this effect was found primarily when considering children’s own evaluations of parental attitudes.

APIMs were constructed for parental and sibling locus of control, with parental positive attitude toward the child used as the mediating variable. Both parental ratings and children’s ratings were considered. Summarizing the results obtained through actor–partner modeling, it can be concluded that perceived parental positive attitude is an important mediator between parental and child locus of control. Parents with a more internal locus of control influence how children evaluate parental attitudes toward them, and in turn, parental attitude is a significant predictor in the development of siblings’ locus of control.

This result supports the widely recognized scientific assumption that subjective perception of events (including those within the family) is the most important environmental factor in the formation of personality traits (Gidziela et al., 2023; Turkheimer & Waldron, 2000). The recognition of the importance of environmental influences as lived and perceived “objective” experience has a long history of study, both in Russian and international psychological traditions (Grishina, 2012).

Maternal locus of control, more often than paternal locus of control, exerts both direct effects on sibling personality characteristics and indirect effects through mediating variables, namely parameters of parental attitudes. The influence of maternal locus of control on siblings within the parental dyad is realized not only as actor effects but also as partner effects, that is, through its impact on paternal attitudes toward the children.

## Conclusion

In today’s rapidly changing world, locus of control is an extremely important personality characteristic that reflects a person’s confidence in their ability to meaningfully influence their own life despite the fast-paced changes around them. Individuals with an internal locus of control show higher levels of mental and physical health, are less vulnerable to stress and depression, and are less prone to deviant behavior.

In the present study, the relationship between parental attitudes and locus of control across two generations of families was examined using actor–partner interdependence modeling. The findings highlight the important role of parental positive attitude as a mediator between parental and child locus of control. Parents with an internal locus of control relate to their children in a more consistent and warm manner, which, in turn, positively influences the development of internality in siblings. The results emphasize the particular significance of perceived parental attitudes in linking parental and child personality characteristics. Maternal locus of control, more often than paternal locus of control, was associated with the corresponding characteristic in children both directly and indirectly — through parameters of parental attitudes — operating as both actor and partner effects.

## Limitations

This study has several limitations. First, its cross-sectional design precludes causal interpretations of the observed associations between parental and child locus of control. Second, the sample was limited to intact families with two children, limiting the generalizability of the findings to other family structures. In addition, the study did not account for socio-economic variables that might influence both parenting practices and children’s locus of control.
